# The *NPR1* ortholog *PhaNPR1* is required for the induction of *PhaPR1* in *Phalaenopsis aphrodite*

**DOI:** 10.1186/1999-3110-54-31

**Published:** 2013-09-06

**Authors:** Jen-Chih Chen, Hsiang-Chia Lu, Cheng-En Chen, Hua-Fang Hsu, Hong-Hwa Chen, Hsin-Hung Yeh

**Affiliations:** 1 Institute of Biotechnology, National Taiwan University, Taipei 106, Taiwan, ROC; 2 Agricultural Biotechnology Research Center, Academia Sinica, Taipei 106, Taiwan; 3 Department of Plant Pathology and Microbiology, National Taiwan University, 1, sec 4, Rooselvet Road, Taipei, 106 Taiwan; 4 Research Center for Plant Medicine, National Taiwan University, Taipei, 106 Taiwan; 5 Department of Life Sciences, National Cheng Kung University, Tainan, 701 Taiwan; 6 Institute of Tropical Plant Sciences, National Cheng Kung University, Tainan, 701 Taiwan; 7 Orchid Research Center, National Cheng Kung University, Tainan, 701 Taiwan

**Keywords:** NPR1, PR1, *Phalaenopsis aphrodite*, Salicylic acid, Systemic acquired resistance

## Abstract

**Background:**

Systematic acquired resistance (SAR) is an effective broad-spectrum defense mechanism that confers long-lasting protection against biotrophic pathogens trough defense related salicylic acid (SA) signaling. Gene(s) involved in SAR have been extensively studied in dicot plants; however, remains largely unresolved in monocot plants. *NPR1*, an evolutionary conserved gene, plays a central role in SAR, and *PR-1* is widely used as a marker for effective SA signaling.

**Results:**

We identified *NPR1* and *PR-1* homologous genes, *PhaNPR1* and *PhaPR1*, from an economically important orchid, *Phalaenopsis aphrodite*, and characterized their roles in SA signaling and *Cymbidium mosaic virus* (CymMV) resistance. A phylogenetic analysis of NPR1 homologs showed that these genes appear to have evolved before angiospermy. Similar to *Arabidopsis NPR1*, *PhaNPR1* was only moderately induced upon SA treatment and CymMV infection. Although *PhaPR1* shows only 36% identity with *AtPR1*, its promoter shared conserved elements with those of other *PR-1* genes, and it was induced upon SA treatment and CymMV infection. After CymMV infection, silencing on *PhaNPR1* also reduced *PhaPR1* expression; however, CymMV accumulation was not affected.

**Conclusions:**

In conclusion, after virus infection, *PhaNPR1* is required for *PhaPR1* induction, but plays little role in defense against CymMV.

**Electronic supplementary material:**

The online version of this article (doi:10.1186/1999-3110-54-31) contains supplementary material, which is available to authorized users.

## Background

Orchidaceae is a widespread monocot family that undergoes rapid speciation. More than 20,000 species in 850 genera have been recorded, and it is believed to be the largest family of angiosperms. Due to adaptive radiation, orchids have evolved an array of strategies to successfully colonize diverse terrestrial ecosystems. Therefore, they provide rich resources to study evolution and the mechanisms of plant-environment interactions. Their diverse flower colors and shapes make them attractive in the floral industry. Orchids are economically important in countries worldwide; however, their production is harmed by pathogen attacks. Our knowledge concerning orchid defense is still limited.

Plants rely on innate immunity to counteract challenges from pathogens. The defense system is tightly regulated and coordinated through several inducible responses involving phytohormones, such as salicylic acid (SA), jasmonic acid (JA), and ethylene (Pieterse et al., 2009). SA is important for resistance against biotrophic pathogens by inducing systemic acquired resistance (SAR), an effective broad-spectrum defense mechanism that confers long-lasting protection. When SAR occurs, several pathogenesis-related genes (*PR* genes) are induced locally at the site of infection and systemically in distal plant tissues (Durrant and Dong, 2004). Studies have demonstrated that many PR proteins have antimicrobial properties (van Loon et al., 2006); however, a single *PR* gene often confers limited resistance to pathogen invasion in transgenic plants. Thus, it is generally believed that the concerted expression of many *PR* genes confers SAR resistance (Durrant and Dong, 2004).

The *PR-1* gene family was the first identified among a host of genes involved in plant defense against pathogens, including oomycetes and fungi. PR-1 proteins are evolutionary conserved among plants, fungi and animals; however, the precise biological functions of PR-1 proteins remain elusive (van Loon et al., 2006). In addition, the expression of *PR-1* genes has been used as a molecular marker to monitor SA signaling and the onset of SAR in various plants, including *Arabidopsis*, tobacco, tomato, rice and barley (van Loon et al., 2006). In *Arabidopsis*, 22 PR-1 paralogs have been identified; however, only one, *AtPR-1* (AT2G14610), is induced by SA, and it has been suggested to be the lone PR-1 protein for induced resistance (van Loon et al., 2006). Unlike *Arabidopsis*, 12 rice PR-1 paralogs were induced upon pathogen attack (Mitsuhara et al., 2008). The regulation of different *PR-1* genes can be diverse; therefore, it is difficult to predict *PR-1* regulation based on sequence similarity.

The induction of *AtPR-1* and other *PR* genes during SAR relies on the expression of a functional NPR1 (non-expressor of pathogenesis-related genes 1), which is a conserved central positive regulator of SA signaling (Durrant and Dong, 2004). Recently, NPR1 was found to serve as a receptor for SA through Cys^521/529^ in *Arabidopsis* (Wu et al., 2012). Mutations in *NPR1* result in breached local basal resistance and a higher accumulation of virulent pathogens, such as *Pseudomonas syringae* pv. *maculicola* (Glazebrook et al., 1996). In contrast, overexpression of NPR1 protein enhances broad-spectrum disease resistance in *Arabidopsis,* rice and wheat, suggesting that the NPR1-mediated defense mechanism is evolutionary conserved across a wide range of species (Cao et al., 1998; Chern et al., 2001; Makandar et al., 2006). In addition, *NPR1* homologs from rice (*OsNPR1*/*NH1*), *Theobroma cacao* (*TcNPR1*), or *Vitis vinifera* (*VvNPR1.1*) were able to complement an *npr1* mutation in *Arabidopsis* (Le Henanff et al., 2011; Shi et al., 2010). However, in contrast to *NPR1* overexpression in *Arabidopsis*, the overexpression of *OsNPR1/NH1* in rice spontaneously activated resistant genes and resulted in a lesion-mimic phenotype (Chern et al., 2005). This result indicates regulation diversities in SAR among different species.

In *Arabidopsis*, the transcription of *NPR1* is only moderately induced upon SA treatment, and post-translational regulation plays a key role in NPR1 activation (Durrant and Dong, 2004). In the uninduced state, NPR1 is present as an oligomer in the cytosol; however, the induction of SAR changes the cellular redox potential of NPR1 and results in its reduction to a monomeric form (Durrant and Dong, 2004). This event results in the accumulation of NPR1 in the nucleus, which interacts with the TGA family of basic leucine zipper (bZIP) transcription factors. The NPR1-TGA complex subsequently induces the expression of defense-related genes, including the *PR* genes (Durrant and Dong, 2004). The cysteine residues Cys^82^ and Cys^216^ in the NPR1 protein are important for the oligomer formation, and the mutation of Cys^150^, Cys^155^, or Cys^160^ leads to a reduction of NPR1 accumulation (Durrant and Dong, 2004). These results indicate that the conserved cysteine residues are important for its regulation at the protein level. Recently, it was shown that the turnover of NPR1 plays dual roles to both prevent and stimulate gene transcription in the regulation of plant immunity (Spoel et al., 2009).

In this study, we aimed to identify key components of SAR, NPR1 and PR1, from a commercially important orchid, *Phalaenopsis aphrodite* subsp. *formosana* and to understand their roles in SA signaling as well as virus defense. Phylogenetic analyses revealed that *PhaNPR1* is an ortholog of *NPR1* while PhaPR1 protein shares only moderate similarity with known PR1-like proteins*.* In spite of low sequence similarity of PhaPR1 to other PR1-like proteins, *PhaPR1* was strongly induced upon SA treatment and virus infection but not JA treatment. The transient knockdown of the *PhaNPR1* suggested that PhaNPR1 may act upstream of *PhaPR1* though defense against CymMV may not require its action. This work provides the basis for further studies of SAR in orchids.

## Methods

### Growth conditions and chemical applications

*Phalaenopsis aphrodite* subsp. *formosana* plants, a commercial orchid variety, were purchased from the Taiwan Sugar Research Institute (Tainan, Taiwan). The plants were maintained in an insect-proof controlled greenhouse with a 12 h photoperiod (200 μmole m^-2^s^-1^) at 25°C. Upon receipt, reverse transcription-polymerase chain reaction (RT-PCR) was conducted to ensure that the orchid plants used were free of virus. The primer pairs, ORSV-CP-F/ORSV-CP-R and CymMV-CP-F/CymMV-CP-R, targeting the coating proteins of two prevalent orchid viruses, ORSV and CymMV, were used (Additional file 1: Table S3). For phytohormone treatment, sodium salicylate (Sigma, 10 mM in water) and methyl jasmonate (Sigma, 45 mM in 1% [v/v] ethanol) were directly sprayed onto the plants. The control plants were sprayed with water or 1% ethanol.

### Identification of PhaNPR1 and PhaPR1

The rapid amplification of cDNA ends (RACE) was performed to obtain 5′ and 3′-end cDNAs of the NPR1 and PR-1 homologs *PhaNPR1 and PhaPR1* from *P. aphrodite* subsp. *formosana* using the SMART-RACE cDNA amplification kit (Clontech; Mountain View, CA, USA) according to the manufacturer’s instructions. The primers used for this study are described in Additional file 1: Table S3. The primer pairs, NPR1F/NPR1R were designed from the *NPR1* conserved region to amplify partial *PhaNPR1* cDNA using RNA isolated from *P. aphrodite* subsp. *formosana* treated with salicylic acid. The amplified fragment was cloned, and the complete sequences were determined. NPR1R was used as a gene-specific primer (GSP) for 1^st^ RACE PCR and NPR1 5' NGSP was used as a 2^nd^ GSP for nested PCR to obtain the 5′ end of *PhaNPR*. The 3′ end of *PhaNPR1* was amplified using NPR1 F and NPR13′ NGSP as 1^st^ and 2^nd^ GSPs for 3′RACE to amplify the 3′ end of *PhaNPR1*. Both RACE amplified 5′ and 3′ ends of *PhaNPR1* were cloned, and the complete sequences were determined. The primer pairs NPR1 ORFF/NPR1 ORFR were designed from the obtained 5′- and 3′- RACE amplified fragments and used in a PCR reaction to obtain the full-length open reading frame (ORF) of *PhaNPR1*. The full-length ORF of *PhaNPR1* was cloned, and the complete sequence was determined. The cloning of *PhaPR1* was performed using essentially the same method as that used to clone *PhaNPR1*, except the primer pair PR1F/PR1R were used in the PCR reaction to obtain partial *PhaPR1* cDNA, PR1 5′ GSP and PR1 5′ NGSP primers were used as1^st^ and 2^nd^ GSPs in the 5′-RACE reaction, PR1 3′ GSP and PR1 3′ NGSP primers were used as 1^st^ and 2^nd^ GSPs in the 3′-RACE reaction, and the entire *PR1* ORF was amplified using the primer pair PR1 ORFF/PR1 ORFR.

### Genomic DNA extraction

Genomic DNA from *P. aphrodite* was purified as previously described (Carlson et al., 1991) with some modifications. Two grams of homogenized leaf tissue were treated with 15 ml of 65°C pre-warmed extraction buffer (100 mM Tris–HCl, 1.4 M NaCl, 20 mM EDTA, 2% CTAB, 1% PVP, 200 μl β-mercaptoethanol) and incubated for 2 h at 65°C. Subsequently, 15 ml of chloroform was added into the extraction mix, and the extraction mixture was mixed at room temperature for 15 min; the mixture was centrifuged at 5,000 × g for 15 min at 4°C. The aqueous phase was filtered through a 70 μm cell strainer (BD Biosciences; Bedford, MA, USA), and the DNA was precipitated using isopropanol. The precipitated DNA was dissolved in 1.3 ml of 1 M NaCl, and treated with RNase to remove the remaining RNA. After RNase treatment, a phenol-chloroform extraction step followed by isopropanol precipitation was conducted to obtain purified DNA.

### Cloning of *PhaPR1* Promoter

The 5′-flanking region of *PhaPR1* was amplified using the GenomeWalker™ Universal kit (Clontech). The genomic DNA from *P. aphrodite* was digested with restriction enzymes, *Dra* I, *EcoR* V, *Pvu* II and *Stu* I, to generate blunt-end fragments, and subsequently the GenomeWalker Adaptors were ligated to the DNA fragments to generated four DNA libraries. The ligated products were used as templates, and the primer pair, Adaptor Primer 1/PR1GSP1 (Additional file 1: Table S3), was used in the primary PCR reaction. A nested PCR reaction using the primer pair, Adaptor Primer 2/PR1GSP2 (Additional file 1: Table S3), was performed to identify the gene-specific 5′-flanking sequence. The PCR products were cloned into a pGEM-T Easy vector (Promega; Madison, WI, USA) and sequenced.

### Infection with *Cymbidium mosaic virus*

A total of 0.5 g of CymMV-infected plant tissue was ground in 300 μl of inoculation buffer (0.05 M NaH_2_PO_4_/Na_2_HPO_4_ pH 7.0) and manually rubbed into carborundum-dusted *Phalaenopsis* leaves with hands wearing latex gloves. The inoculated leaves were washed with excess distilled water.

### Construction of gene silencing vectors and infiltration with *Agrobacteria*

The oligonucleotide pairs PhaNPR1-hpRNA-F1/PhaNPR1-hpRNA-R1 and PhaNPR1-hpRNA-F2/PhaNPR1-hpRNA-R2 (Additional file 1: Table S3) were used to obtain the *PhaNPR1* short hairpin fragments PhaNPR1-hpRNA-1 and PhaNPR1-hpRNA-2, respectively. Each set of primer pairs was mixed, denatured at 72°C for 10 min, and annealed at 25°C for 10 min. Both resulting double-stranded fragments were cloned into the Gateway entry vector pENTR™/D-TOPO® (Invitrogen; Carlsbad, CA, USA) followed by an LR Gateway cloning reaction (Invitrogen) to transfer the fragments into pB7GWIWG2(I) to obtain pB7G-NPR1-1 and pB7G-NPR1- 2. The constructs were transformed into *Agrobacterium tumefaciens* LBA4404 by electroporation. The verified *A. tumefaciens* strains were cultured in 2 ml of YEB medium (5 g/L beef extract, 1 g/L yeast extract, 5 g/L peptone, 5 g/L sucrose, 0.5 g/L MgCl_2_) containing 100 mg/L kanamycin and 100 μM acetosyringone and grown at 28°C for overnight. The next day, 1 ml of the bacterial culture was transferred into 10 ml of YEB medium containing 100 mg/L kanamycin and 100 μM acetosyringone and further incubated at 28°C until reaching an OD_600_ of 1.0~1.2. The *A. tumefaciens* cultures were centrifuged at 3,000 × g for 10 min, and the cells were resuspended in 1 ml of infiltration medium (10 mM MES, 10 mM MgCl_2_, and 100 μM acetosyringone) and incubated at room temperature for 3 h. Each *Phalaenopsis* plant was infiltrated with 100 μl of *A. tumefaciens* suspension.

### RNA isolation, real-time RT-PCR

For RT-PCR, total RNA was extracted from the orchid plants as described (Tian et al., 1996). The RNA was treated with RNase-free DNase (Ambion) to eliminate genomic DNA contamination. Subsequently, 0.5 μg of DNA-free RNA from each sample was used for the synthesis of first strand cDNA using Moloney murine leukemia virus (M-MLV) reverse transcriptase according to the manufacturer’s instructions (Invitrogen). For real-time RT-PCR, 200 ng total RNA treated with TURBO DNA-free kit (Ambion) was used as a template for cDNA synthesis using Moloney murine leukemia virus (MMLV) reverse transcriptase following the manufacturer's instructions (Promega). The cDNA corresponding to 50 ng total RNA was used for real-time PCR using a SYBR Green staining method (ABI StepOne™ Real-Time PCR system, Applied Biosystems). The primers used are listed in Additional file 1: Table S3. PCR products of analyzed genes were sequenced to validate the correct analysis of gene targets. Genes analyzed were from 3 biological replicates and each sample was analyzed for 3 technical replicates. The relative quantification was calculated according to the manufacturer’s instructions (Applied Biosystems). The *Ubiquitin 10* gene was used as an internal quantification control.

### Phylogenetic analysis

Phylogenetic analysis of the *NPR1* genes was conducted using MAGA 5.0 with the maximum likelihood (ML) method. The branch support was estimated using bootstrapping with 1,000 replicates. The sequences used in this study were obtained from NCBI GenBank and are listed in the supplementary information.

## Results

### Sequence analysis of *PhaNPR1* and *PhaPR1*

**Figure 1:**
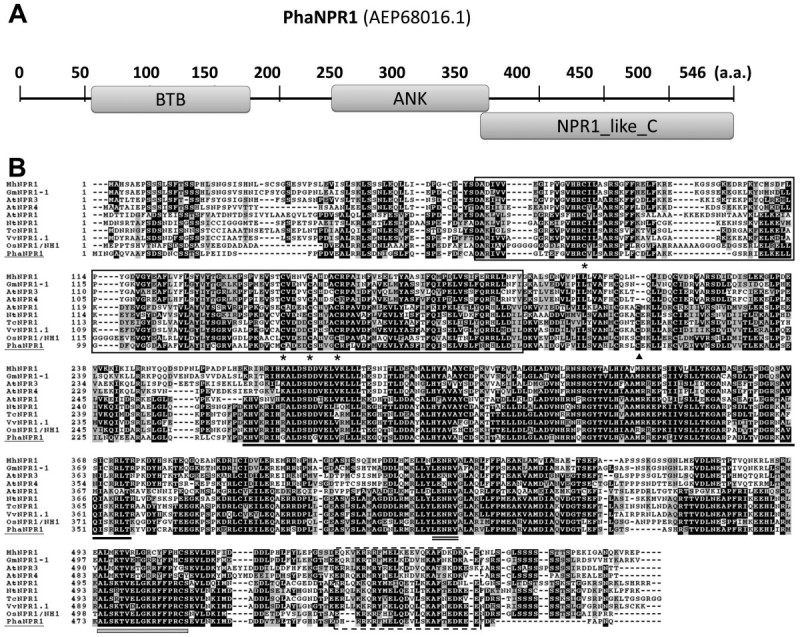
**Protein structures of** ***Phalaenopsis*** **NPR1. (A)** A schematic representation of PhaNPR1. The three major domains, BTB, ankyrin repeats domains (ANK), and C terminal domain (NPR1_like_C), of this protein are indicated with boxes. The two 21-nt regions indicate the regions used for *PhaNPR1* silencing. **(B)** Alignment of PhaNPR1 (underlined) with NPR1 homologs whose localizations have been determined. AtNPR3, AtNPR4 from *Arabidopsis* (Liu et al., 2005; Zhang et al., 2006), MhNPR1 from *Malus hupehensis* (Zhang et al., 2011), GmNPR1-1 from *Glycine max* (Sandhu et al., 2009), VvNPR1.1 from grapevine (Le Henanff et al., 2011), and NtNPR1 from tobacco (Maier et al., 2010), were localized to the nucleus, while AtNPR1 from *Arabidopsis*, TcNPR1 from *Theobroma cacao* (Shi et al., 2010), and OsNPR1/NH1 from rice were localized to the cytoplasm without reductants. The BTB/POZ and ANK domains, and the C-terminal nuclear localization signal (NLS) are indicated with a solid-lined box, underline, and dashed-lined box, respectively. The conserved LENRV motif is indicated with a double line and a NIMIN binding site is labeled with (*). The conserved cysteine residues are indicated with an asterisk (*), and the cysteine residue corresponding to C^216^ in *Arabidopsis*, which is important for the redox regulation of NPR1, is indicated with a black triangle.

**Figure 2:**
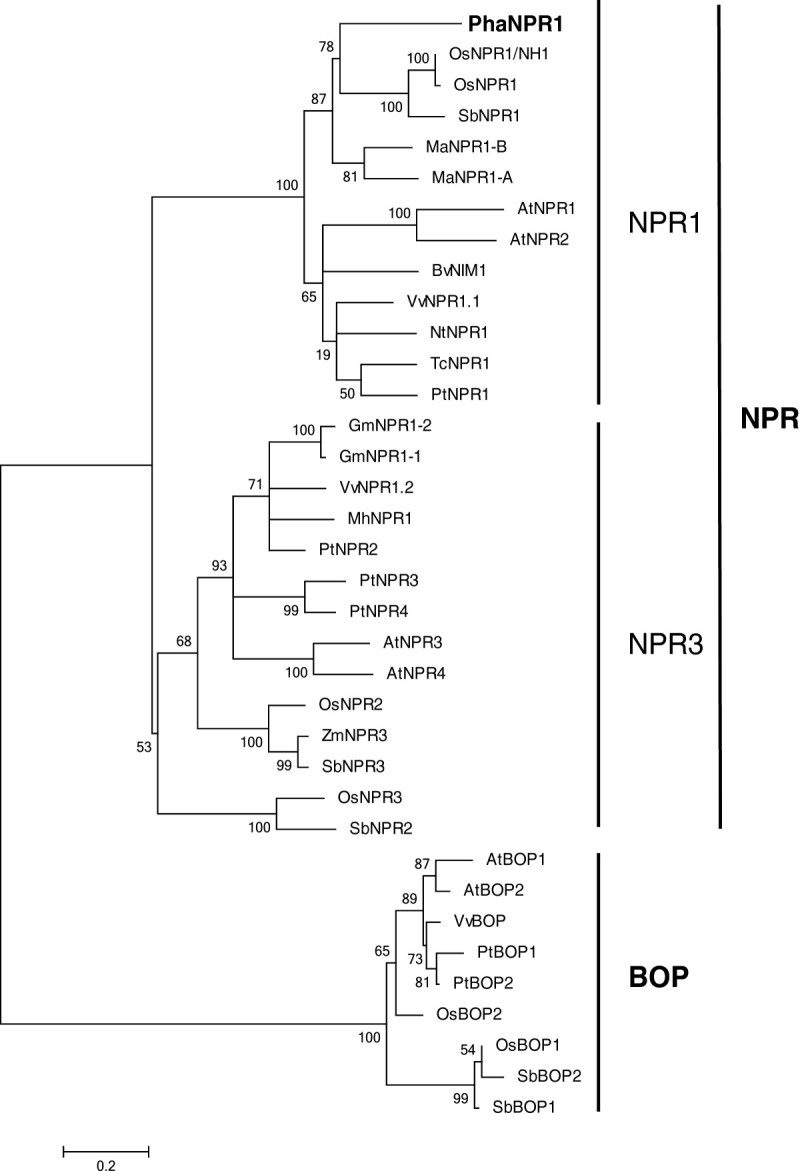
**Phylogenetic tree of the selected NPR1 homologs.** The deduced amino acid sequences of NPR1 homologs from different plant species were used to construct the tree using the maximum likelihood method with 1000 bootstrap values indicated. The information for the homologs used in the analysis is listed in Additional file 1: Table S2. PhaNPR1 is indicated in bold. Members from the five species with reference genomes were included to identify orthologous groups. Three orthologous groups were identified and named NPR1, NPR3, and BOP. Because the NPR1 and NPR3 groups were closely related, these two groups were joined to form a larger NPR group.

To identify *NPR1* and *PR1* homologs in *Phalaenopsis aphrodite* subsp. *formosana*, primers were designed based on the conserved domain sequences to amplify partial sequences of *NPR1* and *PR1*. Subsequently, RACE-PCR was used to obtain full-length transcripts of both genes. The *NPR1* homolog *PhaNPR1* (GenBank accession no. JN630802) was identified, which comprises a full-length 1938 bp sequence encoding a 546 amino acid protein with a predicted molecular weight of 60.9 kDa and pI of 5.4. The protein shows 50% sequence identity with the *Arabidopsis* NPR1 (AT1G64280) and 61% identity with the rice NPR1 (Os01g0194300). The protein also shared conserved domains with other known NPR homologs: a BTB/POZ domain (amino acids 56 to 177), ankyrin repeat domains (amino acids 238 to 369), and a NPR1/NIM1 like defense protein C terminal motif (amino acids 353 to 546) (Figure 1A). A multiple alignment using ClustalX showed that the conserved cysteine residues, including the residues corresponding to AtNPR1 Cys^82^ and Cys^216^, which are required for oligomerization, were also present in PhaNPR1 (Figure 1B). In addition, both motifs required for NIM1-INTERACTING (NIMIN) protein binding, the LENRV motif and a NIMIN binding site, were found in PhaNPR1 (Figure 1B). A phylogenetic analysis of NPR1 proteins using a bootstrap consensus for maximum likelihood (ML) revealed that NPR1 homologs can be classified into three major groups, NPR1, NPR3, and BOP, with high confidence in which both dicot and monocot species have paralogs in each major group (Figure 2). The phylogenetic analysis also indicated that members of the NPR1 and NPR3 gene families are closely related, and might have evolved from an NPR ancestor after the divergence of the *NPR* and *BOP* genes prior to the evolution of angiospermy (Figure 2). We therefore proposed the classification of NPR homologs into NPR and BOP clades; the NPR clade is further divided into two NPR1 and NPR3 subclades. PhaNPR1 was clearly grouped into the NPR1 subclade with other members from monocot plants, including *Oryza* and *Musa acuminata* (Figure 2).

**Figure 3:**
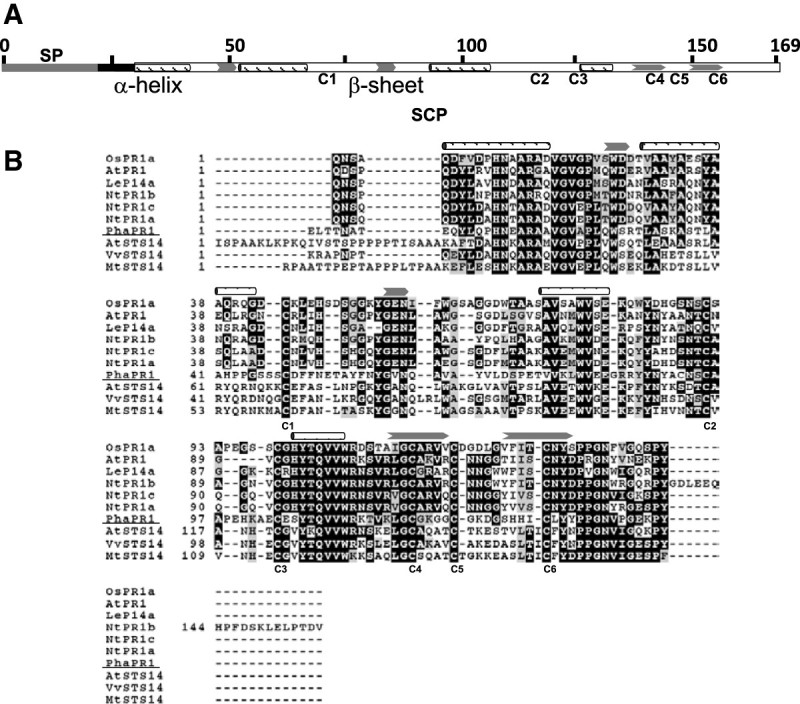
**The structure of** ***Phalaenopsis*** **PR1. (A)** A schematic diagram of PhaPR1. The full-length, 169 amino acid protein contains a predicted signal peptide (SP) of 21 residues in length indicated in gray, the 4 α-helixes are indicated (), and the 4 β-sheets are also shown (). The 6 conserved cysteine residues are also indicated with C1-C6. **(B)** Alignment of PhaPR1 (underlined) with PR-1-like proteins, which have antimicrobial functions, and STS14-like proteins. The mature protein sequences were used for the analysis. Their accession numbers are OsPR1a (NP_001058815), AtPR1 (AT2G14610), LeP14a (NP_001234314), NtPR1a (CAA31233), NtPR1b (CAA47374), NtPR1c (CAA35666), AtSTS14 (AT5G66590), VvSTS14 (XP_003635089), and MtSTS14 (XP_003611867).

The *PR1* homolog, *PhaPR1* (GenBank accession no. JX137044), is 767 bp sequence encoding a 169 amino acid protein containing a 21 amino acid signal peptide, as predicted by SignalP, and a SCP domain, which is a conserved domain present in most PR1-like proteins. The predicted molecular weight and pI of the mature protein are 16.2 kDa and 6.95, respectively. The conserved features, which include four α-helices, four β-strands and six conserved cysteine residues, were present in PhaPR1 (Figure 3). However, this protein only shares moderate sequence identities with PR-1 proteins with antimicrobial activity. The identities to tomato P14a (NP_001234314), *Arabidopsis* PR1 (AT2G14610), rice PR-1a (Os07g0129200), and tobacco PR-1a (CAA31233) are 40%, 36%, 35%, and 35%, respectively. PhaPR1 is closely related to STS14-like proteins (~47% identity), which were first identified in the potato and highly expressed in the pistil (Van Eldik et al., 1996). However, the function of these proteins has not been characterized. PhaPR1 is most similar to a PR-1 like protein in *Arabidopsis*, AtSTS14 (AT5G66590), with 43% identity. Although detailed characterizations of these proteins have not been attempted, a search for microarray studies on these genes using Genevestigator revealed that AT5G66590 was induced upon infection of the fungal pathogen *Alternaria brassicicola* (Additional file 1: Table S1).

### Analysis of cis-acting elements in the *PhaPR1* promoter

A 2.5-kb upstream region from the translation start site of *PhaPR1* was identified within the genomic DNA of *P. aphrodite* using genome walking. The sequence was analyzed using web-based *cis*-acting element analysis programs, such as PLACE and PlantCARE. The typical CAAT and TATA boxes are present within 150 bp upstream of the ATG start codon in the promoter region. We identified conserved elements within the 5′-end sequence flanking the promoter region in *PhaPR1* and *AtPR1* (Table 1). The 815-bp upstream promoter region of *AtPR1* is required for the induction response upon SA treatment (Lebel et al., 1998). In this region, several conserved motifs were identified, including a NF-κB binding motif (LS10), an ATATTCTT motif (LS9), which was also identified in tobacco *PR-1a* and *PR-2d* promoters, a bZIP transcription factor binding motif (LS7), and a zinc-finger motif (LS4). The LS10 and LS7 motifs are essential for SAR-mediated *AtPR1* regulation (Lebel et al., 1998). In the *PhaPR1* promoter region, a CGGCATTTCC motif, which is similar to LS10 (GGACTTTTC), at position -440 to -430, a LS9 motif at position -1125 to -1117, and two LS4 motifs at positions -647 to -641 and -1011 to -1005 were identified; however, the LS7 motif was not located within the promoter region (Table 1). We did find an ASF1 motif (TGACG) at positions -294 to -289. This motif was shown to mediate auxin- and salicylic acid-inducible transcription in tobacco TGA transcription factors (Niggeweg et al., 2000). Other consensus motifs identified within the promoter region include PyTGTCNC and several W-box motifs (Table 1). The PyTGTCNC motif has been identified in the promoter region of many *PR* genes, and it is the binding site of the transcription repressor silencing element-binding factor (SEBF) (Boyle and Brisson, 2001). The W-box is a binding motif that has been identified in WRKY transcription factors. The motif is located within the promoter region of many *PR* genes and was also identified in the promoter region of the *Arabidopsis NPR1* gene; this motif is important for the induction of these genes (Yu et al., 2001).

**Table 1:** **Conserved sequence of various motifs in the** ***PhaPR1*** **promoter region**

Motif	Consensus sequence	Sequence in ***PhaPR1*** promoter	Position	Reference
LS9	ATATTCTT	ATATTCTT	-1125 to -1117	(Lebel et al., 1998)
GT-1	GAAAAA	GAAAAA	-1096 to -1091	(Park et al., 2004)
SEBF	PyTGTCNC	GCTGTCAC	-636 to -630	(Boyle and Brisson, 2001)
W-box	(T)(T)TGACY	TTGACT	-1011 to -1006	(Rushton et al., 1996)
		TTTGACT	-648 to -642	
		TTGACC	-459 to -454	
NF-κB	GGGACTTTTCC	CGGCATTTCC	-440 to -430	(Baeuerle and Baltimore, 1996)
ASF1	TGACG	TGACG	-294 to -289	(Katagiri et al., 1989)

### Virus infection and treatment with SA, but not JA, induce the expression of *PhaNPR1* and *PhaPR1*

**Figure 4:**
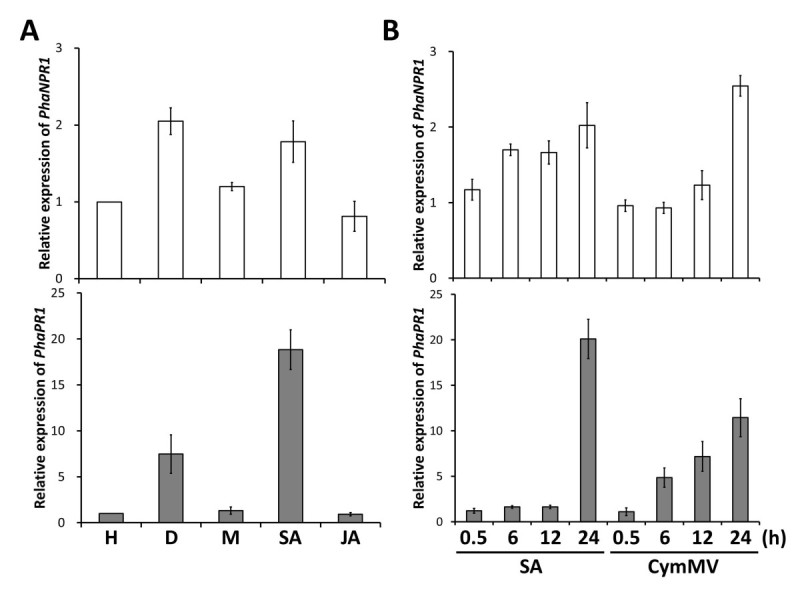
**Changes in the expression of** ***PhaNPR1*** **and** ***PhaPR1*** **under hormone treatments or virus infection. (A)** Analyses of *PhaNPR1* and *PhaPR1* expression in *Phalaenopsis aphrodite* subsp. *formosana* leaves after treatment: (H) Healthy, (D) CymMV infection, (M) 1% [v/v] ethanol, (SA) Sodium salicylate and (JA) methyl jasmonate. **(B)** Analyses of *PhaNPR1* and *PhaPR1* expression at various time intervals after SA treatment or CymMV infection. Data are the mean ± SD of 3 biological replicates. Expression fold changes are relative to that of healthy controls with *UBIQUITIN* level as an internal reference. h: treatment hours.

Although *NPR1* transcripts are only moderately induced upon SA treatment in *Arabidopsis*, the regulation of SA signaling through NPR1 can be different among different species. We determined whether SA treatment could affect the expression of *PhaNPR1*. Similar to *Arabidopsis NPR1*, the *PhaNPR1* transcript was detected in healthy untreated plants, and moderately induced after treatment with SA and CymMV inoculation for 24 h (Figure 4A). A time course of the increased *PhaNPR1* expression under SA treatment and CymMV was subsequently conducted, and the accumulation of *PhaNPR1* transcripts was detected after 6 h of SA treatment but was delayed for 6 h for CymMV inoculated leaves, and the transcript levels of both groups reached to ~2 fold of the control level after 24 h. Treatment with SA and CymMV infection also induced the accumulation of *PhaPR1* transcripts in a manner similar to that of *PhaNPR1* (Figure 4). Notably, treatment with JA did not induce the accumulation of *PhaNPR1* and *PhaPR1* transcripts (Figure 4A).

### Knockdown of *PhaNPR1* reduces the accumulation of *PhaPR1* transcripts but does not affect CymMV concentration

**Figure 5:**
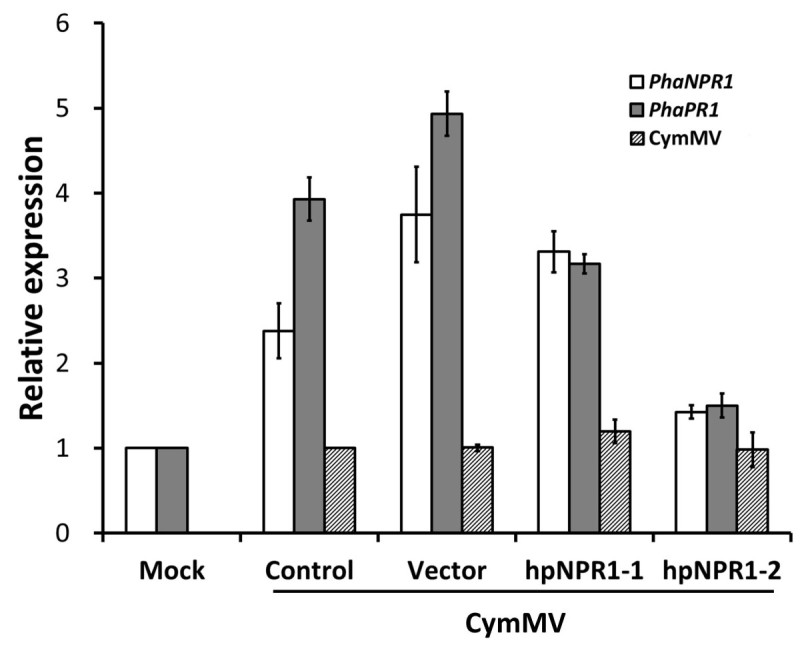
**Changes in** ***PhaPR1*** **expression and CymMV accumulation when** ***PhaNPR1*** **is silenced.** *PhaNPR1* expression was transiently knocked down in the leaves of *P. aphrodite* subsp. *formosana* using *Agrobacteria* carrying short hairpin constructs. The two constructs used carry hpNPR1-1 and hpNPR1-2, respectively, and the leaves of the control group were only with CymMV infection and vector groups were inoculated with *Agrobacteria* carrying the empty vector. The mock treatment was inoculation of 1% [v/v] ethanol. The RNA samples were collected at seven days post inoculation. Data are the mean ± SD of 3 biological replicates. Expression fold changes are relative to that of the mock treatment for *PhaPR1* and *PhaNPR1*, and to that of the control for CymMV concentration with *UBIQUITIN* level as an internal reference.

Our results suggested that NPR1 may play an important role in SA-mediated resistance, and both *PhaNPR1* and *PhaPR1* are induced upon SA treatment and virus infection; therefore, we transiently knocked down the expression of *PhaNPR1* using short hairpin sequences to examine whether it is required for *PhaPR1* transcript accumulation during virus infection and resistance against CymMV. Indeed, after CymMV infected leaves of *P. aphrodite* were transiently inoculated for seven days with *Agrobacteria* containing a *PhaNPR1* short hairpin sequence, the transcript abundance of *PhaNPR1* was suppressed, and coincidentally, the transcript level of *PhaPR1* was also reduced, especially when using the hpNPR1-2 fragment (Figure 5). However, the suppression of *PhaNPR1* expression did not result in over-accumulation of CymMV (Figure 5).

## Discussion

Using PCR, we isolated the full-length cDNA of *NPR1* and *PR1* homologs from a commercially important orchid, *Phalaenopsis aphrodite* subsp. *formosana*. We also identified the promoter sequence of the isolated *PR1* gene, *PhaPR1*. We obtained several interesting findings from the characterization of both genes. First, the phylogenetic analysis of NPR1 homologs from various monocot and dicot species revealed that NPR1 evolved prior to the development of angiospermy, and currently, there are three major groups of NPR1 homologs in every angiosperm species examined thus far (Figure 2). The three phylogenetic groups of *Arabidopsis* NPR1-like proteins share two conserved domains, BTB/POZ and ankyrin repeats, which are responsible for protein-protein interactions; however, BOP1 and BOP2 lack a conserved nuclear localization signal and have shorter C termini (Hepworth et al., 2005). In *Arabidopsis*, proteins in the NPR clade have been demonstrated to be important in plant defense, while proteins in the BOP clade are responsible for plant morphogenesis (Cao et al., 1997; Hepworth et al., 2005; Liu et al., 2005; Zhang et al., 2006). However, all NPR1 paralogs might also share redundant functions in SA perception. The growth of plants can be suppressed through treatment with benzothiadiazole (BTH), an analogue of SA, while mutations in *NPR1* reduce the suppression effect due to impaired SA perception. However, the growth suppression effect was reduced with mutations in other NPR1 paralogs (Canet et al., 2010). It has recently demonstrated that NPR1, NPR3, and NPR4 are receptors of SA in *Arabidopsis*, and the binding of NPR3 and NPR4 to SA is important for their role in NPR1 degradation (Fu et al., 2012; Wu et al., 2012). The double *bop1 bop2* mutant, however, did not show defects in plant resistance, suggesting that BOP proteins might not participate in plant defense systems (Hepworth et al., 2005). Although proteins in the NPR3 subgroup were shown to be involved in plant defense, their functions in plant resistance were contradictory. In *Arabidopsis*, NPR4 plays a positive role against *P. syringe* (Liu et al., 2005). However, a negative role in regulation of *PR* genes was later proposed (Zhang et al., 2006). Moreover, VvNPR1.2, which was classified in the NPR3 subgroup (Figure 2) in grape, could not compliment the function of *Arabidopsis* NPR1 (Le Henanff et al., 2011), suggesting a different function for proteins in the NPR3 subgroup. However, two closely related proteins of VvNPR1.2, GmNPR1-1 and GmNPR1-2 (Figure 2) from soybean, complimented the *Arabidopsis npr1-1* mutant and were considered as functional orthologs of *Arabidopsis* NPR1 (Sandhu et al., 2009). It is possible that different functions have been assigned for evolutionary conserved proteins in different species, but more evidence is needed to obtain a final conclusion.

The redox activity of AtNPR1 is a special post-translational regulation of this important protein, and both Cys^82^ and Cys^216^ in the protein sequence are required for its regulation (Mou et al., 2003). The alignment of NPR1 homologs revealed that the Cys^82^ is conserved among all NPR1 homologs, while the Cys^216^ is conserved only within the NPR1 subgroup (Figure 1B). Indeed, proteins in both the NPR3 and BOP clades, such as AtBOP1, AtNPR4, AtNPR3, MhNPR1, have been localized to the nucleus without presence of reductants (Hepworth et al., 2005; Liu et al., 2005; Zhang et al., 2011; Zhang et al., 2006). In addition to the conserved Cys residues, two regions important for protein interaction with NIMIN proteins are conserved among all NPR1 subclade members including PhaNPR1 (Figure 1B)(Maier et al., 2011). NIMIN proteins are another class of NPR1 interacting proteins, which were first identified with a NPR1 bait, and the interaction plays a regulatory role on activation of NPR1 (Weigel et al., 2001).

The NPR1 homolog from *Phalaenopsis aphrodite* was classified into the NPR1 subgroup and contains cysteine residues corresponding to Cys^82^ and Cys^216^ in AtNPR1 (Figure 1). This result indicates that PhaNPR1 is a *bona fide* ortholog of AtNPR1, and redox activation might also be a part of its regulation machinery. In addition, transcriptional regulation may also be important for PhaNPR1, as its transcript levels correlated well with *PhaPR1* transcript abundance upon SA treatment or challenge with CymMV though *PhaNPR1* was only moderately induced by stimuli (Figure 4). We showed that knocking down *PhaNPR1* transcription resulted in the reduction of *PhaPR1* expression (Figure 5). This result is consistent with our hypothesis that the transcriptional regulation of *PhaNPR1* is important for defense in *Phalaenopsis* and also demonstrates that SA-induced defense may occur through PhaNPR1-mediated pathways.

The PhaNPR1 regulated PR1-like protein, PhaPR1, is much more similar to the *Arabidopsis* STS14-like protein (AT5G66590), whose function has not been characterized, than to *Arabidopsis* PR1 (AT2G14610), which is the only SA inducible PR1-like protein in *Arabidopsis*. However, sequence similarity is not a reliable method for the prediction of protein function; therefore, we identified the promoter region of *PhaPR1* and analyzed whether the *PhaPR1* can be induced upon SA treatment and virus infection. The promoter region contains many conserved motifs, including an ASF1 motif (important for auxin- and salicylic acid-inducible transcription), an LS10-like motif, and many W-box motifs (potential WRKY transcription factor binding site), which are found in promoter of other *PR-1* genes (Table 1). This finding indicates that *PhaPR1* might be regulated through machinery similar to that regulating other well-known PR1 proteins. Indeed, subsequent to the induction of *PhaNPR1* upon SA treatment and CymMV infection, *PhaPR1* was also induced (Figure 4). The expression of *PhaPR1* transcripts was reduced when the transcript level of *PhaNPR1* was down-regulated (Figure 5). Therefore, PhaPR1 could be a downstream factor in the PhaNPR1-mediated defense pathway. However, the concentrations of CymMV were not altered following *PhaNPR1* silencing in CymMV infected plants (Figure 5). *P. aphrodite* infected with CymMV did not show visible symptoms, and still no visible symptoms were observed following *PhaNPR1* silencing (data not shown). The role of NPR1 in viral resistance is often contradicted among different plant species. NPR1-dependent pathway is required for proper N-mediated resistance to *Tobacco mosaic virus* (TMV) in tobacco (Liu et al., 2002). However, in *Arabidopsis*, resistance to *Turnip crinkle virus* (TCV) seems required SA but independent to NPR1 because resistance to TCV was not compromised in both *npr1-1* and *npr1-5* mutant backgrounds (Kachroo et al., 2000). Furthermore, susceptibility of transgenic rice with ectopic expression of AtNPR1 to *Rice yellow mottle virus* (RYMV) was increased without alteration on virus concentration (Quilis et al., 2008). It is likely that similar to resistance to TCV in *Arabidopsis*, resistance to CymMV in *P. aphrodite* goes through a PhaNPR1-independent pathway. However, we have recently identified a Ring-finger domain containing protein, PhaTF15, and its expression is important for CymMV-induced expression of *PhaNPR1* and *PhaPR1*, and also for resistance to the virus. When expression of *PhaTF15* was silenced, the expression of both PhaNPR1 and PhaPR1 were reduced and CymMV accumulated to a high level (Lu et al., 2012). Therefore, a pathway that mediates the expression of *PhaNPR1* but not downstream of *PhaNPR1* may be important to defense against CymMV in *P. aphrodite*.

## Conclusion

In conclusion, we have identified important components of SAR in orchids and showed that unlike the regulation of *Arabidopsis NPR1*, the transcriptional regulation of *PhaNPR1* plays a pivotal role in the activation of SAR. We also showed that PhaPR1 is a downstream component of PhaNPR1-mediated SAR and have identified the promoter region in the *PhaPR1* gene. Additional experiments to identify other elements in the promoter region of *PhaPR1* for PhaNPR1-mediated induction are underway.

## Electronic supplementary material

Additional file 1: Table S1: Differences in the expression of *PR-1*-like genes under pathogen infection or hormone treatment in *Arabidopsis thaliana* (Data extracted from GENEVESTIGATOR). **Table S2**. NPR1 homologs used for phylogenetic analysis. **Table S3**. Primers used in this study. (DOC 144 KB)

Below are the links to the authors’ original submitted files for images.

Authors’ original file for figure 1

Authors’ original file for figure 2

Authors’ original file for figure 3

Authors’ original file for figure 4

Authors’ original file for figure 5

## Abbreviations

BOP: BLADE-ON-PETIOLE
BTH: benzo-(1,2,3)-thiadiazole-7-carbothioic acid S-methyl ester
CymMV: *Cymbidium mosaic virus*
GSP: Gene-specific primer
NPR1: NONEXPRESSOR OF PATHOGENESIS-RELATED GENES1
ORF: Open reading frame
PR-1: PATHOGENESIS-RELATED GENES -1
RACE: Rapid amplification of cDNA ends
SAR: Systematic acquired resistance.
