# Supplementary material for: The NPR1 ortholog PhaNPR1 is required for the induction of PhaPR1 in Phalaenopsis aphrodite
Source: Bot Stud. 2013 Sep 6;54:31. doi: 10.1186/1999-3110-54-31 (PMC5432770; doi:10.1186/1999-3110-54-31)
Supplement: Supplementary file 1 — Additional file 1: Table S1: Differences in the expression of PR-1-like genes under pathogen infection or hormone treatment in Arabidopsis thaliana (Data extracted from GENEVESTIGATOR). Table S2. NPR1 homologs used for phylogenetic analysis. Table S3. Primers used in this study. (DOC 144 KB) [file 40529_2013_31_MOESM1_ESM.doc]

**Supplementary Table I.** Differences in the expression of *PR-1*-like genes under pathogen infection or hormone treatment in *Arabidopsis thaliana* (Data extracted from GENEVESTIGATOR)

|  | Fungi | | | | | bacteria | | virus | | hormone | | | | |
| --- | --- | --- | --- | --- | --- | --- | --- | --- | --- | --- | --- | --- | --- | --- |
|  | A | B1 | B2 | E | P1 | | P2 | C | T | ACC | ETH | JA | SA | BTH |
| AT2G14610  (*AtPR1*) | 2.85 | 1.22 |  | 3.34 | 2.85 | | 1.7 | 6.82 | 3.73 | 1.38 |  |  | 2.1 | 3.83 |
| AT2G14580  (*AtPRB1*) |  |  |  |  | 2.24 | |  | 1.85 |  |  |  |  |  | 1.26 |
| AT4G33720 |  |  |  |  |  | |  |  |  | 1.41 |  |  |  |  |
| At3G19690 |  |  |  |  |  | |  |  |  |  |  |  |  |  |
| AT5G26130 |  |  |  |  |  | |  |  |  |  |  |  |  |  |
| AT4G33730 |  |  |  |  |  | | -1.23 |  |  |  |  |  | -1.24 |  |
| At2G19990 |  |  |  |  |  | |  |  |  |  |  |  |  |  |
| At4G25790 |  |  |  |  |  | |  |  |  |  |  |  | -1.04 |  |
| AT4G33710 |  |  |  |  |  | |  |  |  |  |  |  |  |  |
| AT1G50060 |  |  |  |  |  | |  |  |  |  |  |  |  |  |
| AT4G07820 |  |  |  |  |  | |  |  |  |  |  |  |  |  |
| AT1G01310 |  |  |  |  |  | |  |  |  |  |  |  |  |  |
| AT4G30320 |  |  |  |  |  | |  |  |  |  |  |  |  |  |
| AT4G31470 |  |  |  |  |  | |  |  |  |  |  |  |  |  |
| AT1G50050 |  |  |  |  |  | |  |  |  |  |  |  |  |  |
| AT3G09590 |  |  |  |  |  | |  |  |  |  |  |  |  |  |
| AT4G25780 |  |  | -1.13 |  | -1.84 | | -3.25 |  |  |  |  |  | -1.72 |  |
| AT5G02730 |  |  |  |  |  | |  |  |  |  |  |  |  |  |
| AT2G19970 |  |  |  |  |  | |  |  |  |  |  |  |  |  |
| AT2G19980 |  |  |  |  |  | |  |  |  |  |  |  |  |  |
| AT5G66590  (*AtSTS14*) | 3.35 |  |  |  | -2.53 | | -3.59 | -1.11 |  |  | -1.22 |  |  |  |

There are 22 *PR-1*-like genes in the *Arabidopsis* genome. AT5G57625 was not included here because its probe was lacked in the Affymetrix array. The data shows a log2 value of treatment against the control. Only the numbers >1 are indicated with red to show the up-regulated values and numbers <-1 are indicated in green to show the down-regulated values.

A: *Alternaria brassicicola*, B1: *Botrytis cinerea*, B2: *Blumeria graminis*, E: *Erysiphe orontii*, P1: *P. syringae* pv. Maculicola, P2: *P. syringae* pv. Syringae, C: CaLCuV, T: TuMV, ACC: 1-aminocyclopropane-1-carboxylic acid, ETH: ethylene, JA: jasmonic acid, SA: salicylic acid, and BTH: benzo-(1,2,3)-thiadiazole-7-carbothioic acid S-methyl ester.

**Supplementary Table** II. NPR1 homologs used for phylogenetic analysis.

| **Name** | **Organism** | **GenBank Accession Numbers** | **Reference** |
| --- | --- | --- | --- |
| PhaNPR1 | *Phalaenopsis aphrodite* | AEP68016 | This study |
| AtNPR1 | *Arabidopsis thaliana* | AT1G64280 |  |
| AtNPR2 |  | AT4G26120 |  |
| AtNPR3 |  | AT5G45110 |  |
| AtNPR4 |  | AT4G19660 |  |
| AtBOP1 |  | AT3G57130 |  |
| AtBOP2 |  | AT2G41370 |
| VvNPR1.1 | *Vitis vinifera* | CAO65332 |  |
| VvNPR1.2 |  | XP_002274045 |
| VvBOP |  | CAO23333 |
| BvNIM1 | *Beta vulgaris* | AAT57640 |  |
| NtNPR1 | *Nicotiana tabacum* | ABH04326 |  |
| TcNPR1 | *Theobroma cacao* | ADI24348 |  |
| PtNPR1 | *Populus trichocarpa* | XP_002308281 |  |
| PtNPR2 |  | XP_002322351 |  |
| PtNPR3 |  | XP_002300863 |  |
| PtNPR4 |  | XP_002307566 |  |
| PtBOP1 |  | XP_002323261 |  |
| PtBOP2 |  | XP_002308905 |  |
| OsNPR1/NH1 | *Oryza sativa* Indica | AAX18700 |  |
| OsNPR1 | *Oryza sativa* Japonica | AAP92751 |  |
| OsNPR2 |  | NP_001044363 |  |
| OsNPR3 |  | NP_001050850 |  |
| OsBOP1 |  | NP_001065717 |  |
| OsBOP2 |  | ABE11621 |  |
| ZmNPR3 | *Zea mays* | NP_001147587 |  |
| MaNPR1-B | *Musa acuminata* | ABI93182 |  |
| MaNPR1-A |  | ABL63913 |
| SbNPR1 | *Sorghum bicolor* | XP_002455011 |  |
| SbNPR2 |  | XP_002464110 |  |
| SbNPR3 |  | XP_002456404 |  |
| SbBOP1 |  | XP_002442682 |  |
| SbBOP2 |  | XP_002450246 |  |
| MhNPR1 | *Malus hupehensis* | ADP95762 |  |
| GmNPR1-1 | *Glycine max* | NP_001238658 |  |
| GmNPR1-2 |  | NP_001238674 |  |

The genomes of *Arabidopsis thaliana*; *Vitis vinifera*; *Populus trichocarpa*, *Oryza sativa*, and *Sorghum bicolor* have been sequenced, and all of the NPR1 homologs from these organisms were extracted for analysis.

**Supplementary Table** III. Primers used in this study

| Primer | Sequence | Description |
| --- | --- | --- |
| NPR1F | 5’-AGAGATACACGGTGCTTCATGTTG-3’ | qPR1 of NPR1  NPR1 cloning and 3’RACE |
| NPR1R | 5’-GCCACACCAACTCTATTTTCAAG-3’ | qPR1 of NPR1  NPR1 cloning and 5’RACE |
| NPR1 5' NGSP | 5’-GGAGAAGGCTTCCCTTCTTCCGTCG-3’ | NPR1 5’RACE |
| NPR1 3' NGSP | 5’- CGACGGAAGAAGGGAAGCCTTCTCC-3’ | NPR1 3’RACE |
| NPR1 ORFF | 5’-ATGATTAACGGTGCGCAAGT-3’ | Clone NPR1 ORF |
| NPR1 ORFR | 5’-CTATTGATTCGGAGCAAACTT-3’ | Clone NPR1 ORF |
| PR1F | 5’-AGGACCCTGGCGTCTAAAG- 3’ | qPR1 of PR1 |
| PR1R | 5’-TATTACAAATCAAACCGCTAAAG- 3’ | qPR1 of PR1 |
| PR1 5' GSP | 5’-TATTACAAATCA AACCGCTAAAG-3’ | PR1 5’RACE |
| PR1 5' NGSP | 5’-ATGGTGGGAACCATTCTTTACCACAG-3’ | PR1 5’RACE |
| PR1 3' GSP | 5’-AGGACCCTGGCGTCTAAAG-3’ | PR1 3’RACE |
| PR1 3' NGSP | 5’-TACGTACTCGACTCGCCGGAGACGG-3’ | PR1 3’RACE |
| Adaptor Primer 1 | 5’-GTAATACGACTCACTATAGGGC-3’ | PR1 promoter cloning |
| PR1GSP1 | 5’-GAGCACGTAGGCCACAGCTTGATTGACT-3’ | PR1 promoter cloning |
| Adaptor Primer 2 | 5’- ACTATAGGGCACGCGTGGT -3’ | PR1 promoter cloning |
| PR1GSP2 | 5’- GTTGTGGGGCTGAAGGTACTGCTCCGT-3’ | PR1 promoter cloning |
| PhaNPR1-hpRNA-F1 | 5'-CACCCGAGATCATTGATTCCTTCCC-3' | Construction of hairpin NPR1 expression vector |
| PhaNPR1-hpRNA-R1 | 5'-GGGAAGGAATCAATGATCTCG-3' | Construction of hairpin NPR1 expression vector |
| PhaNPR1-hpRNA-F2 | 5'-CACCCTCCGAAGATCATCGACGAAA-3' | Construction of hairpin NPR1 expression vector |
| PhaNPR1-hpRNA-R2 | 5'-TTTCGTCGATGATCTTCGGAG-3' | Construction of hairpin NPR1 expression vector |
| PhaNPR1 qPCR F | 5'-AAGGAGCAAGGCCAGCTGAT-3' | qPCR of NPR1 |
| PhaNPR1 qPCR R | 5'-TCTTCCGTCGCCCTACAGTAA-3' | qPCR of NPR1 |
| PhaPR1 qPCR F | 5'-GGATCATCGTCTTGCGATTT-3' | qPCR of PR1 |
| PhaPR1 qPCR R | 5'-CCGCACAACTGTTACATGCAT-3' | qPCR of PR1 |
| CymMV qPCR F | 5’-TGATGCTGGCCACTAACGATC-3’ | qPCR of CymMV CP |
| CymMV qPCR R | 5’-GGAATCAACGGCATCGAAGA-3’ | qPCR of CymMV CP |
| ubiquitin qPCR F | 5’-CCGGATCAGCAAAGGTTGA-3’ | qPCR of ubiquitin |
| ubiquitin qPCR R | 5’-AAGATTTGCATCCCTCCCC-3’ | qPCR of ubiquitin |
| CymMV-CP-F | 5'-GAAATAATCATGGGAGAGCC-3' | Virus detection |
| CymMV-CP-R | 5'- AGTTTGGCGTTATTCAGTAGG -3' | Virus detection |
| ORSV-CP-F | 5'-ACGCACAATCTGATCCGTA-3' | Virus detection |
| ORSV-CP-R | 5'-ATCCGCAGTGAAAACCC-3' | Virus detection |
| PR1 ORFF | 5'-ATGGCTAAACAGATGCAGTTATG-3' | PR1 ORF cloning |
| PR1 ORF R | 5'-TCAATAAGGTTTCTCGCCAGGAAC-3' | PR1 ORF cloning |
