# Supplementary figures and images for: The NPR1 ortholog PhaNPR1 is required for the induction of PhaPR1 in Phalaenopsis aphrodite
Source: Bot Stud. 2013 Sep 6;54:31. doi: 10.1186/1999-3110-54-31 (PMC5432770; doi:10.1186/1999-3110-54-31)

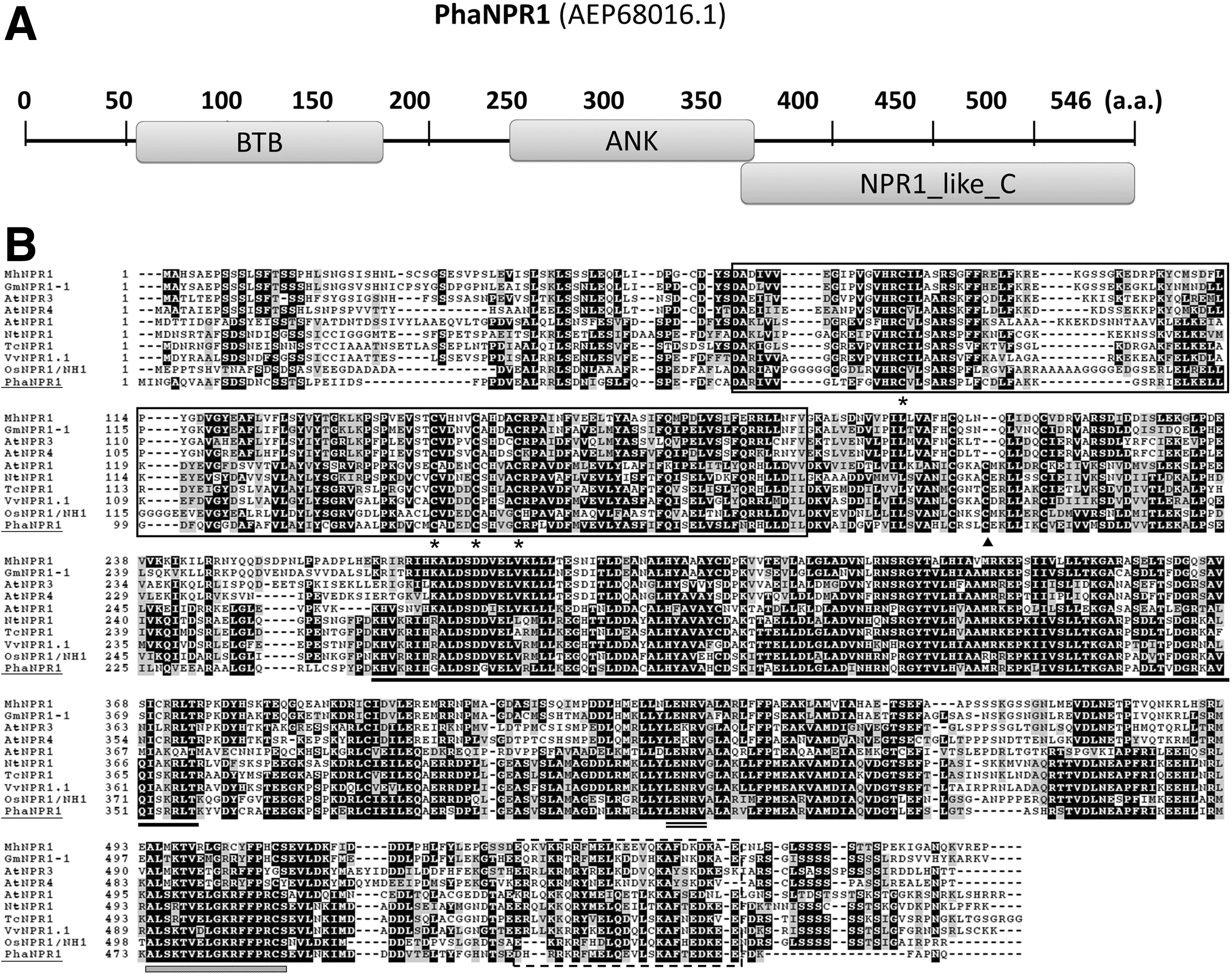

Supplement: Supplementary file 2 — Authors’ original file for figure 1 [file 40529_2013_31_MOESM2_ESM.tif]

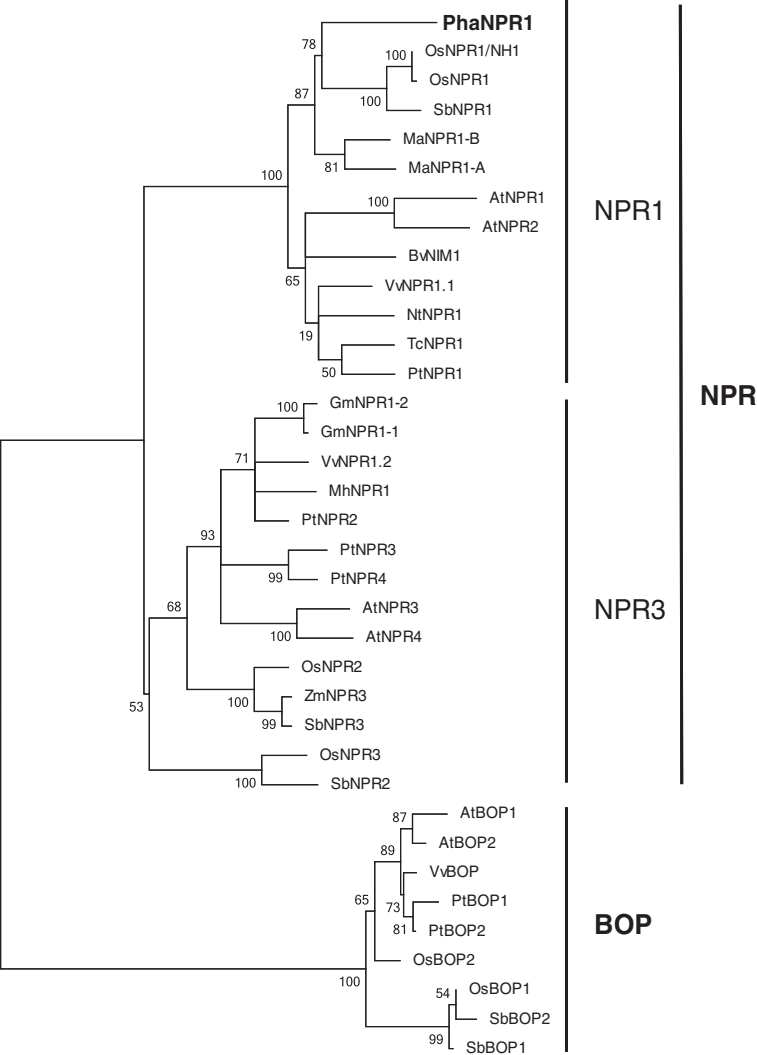

Supplement: Supplementary file 3 — Authors’ original file for figure 2 [file 40529_2013_31_MOESM3_ESM.pdf]

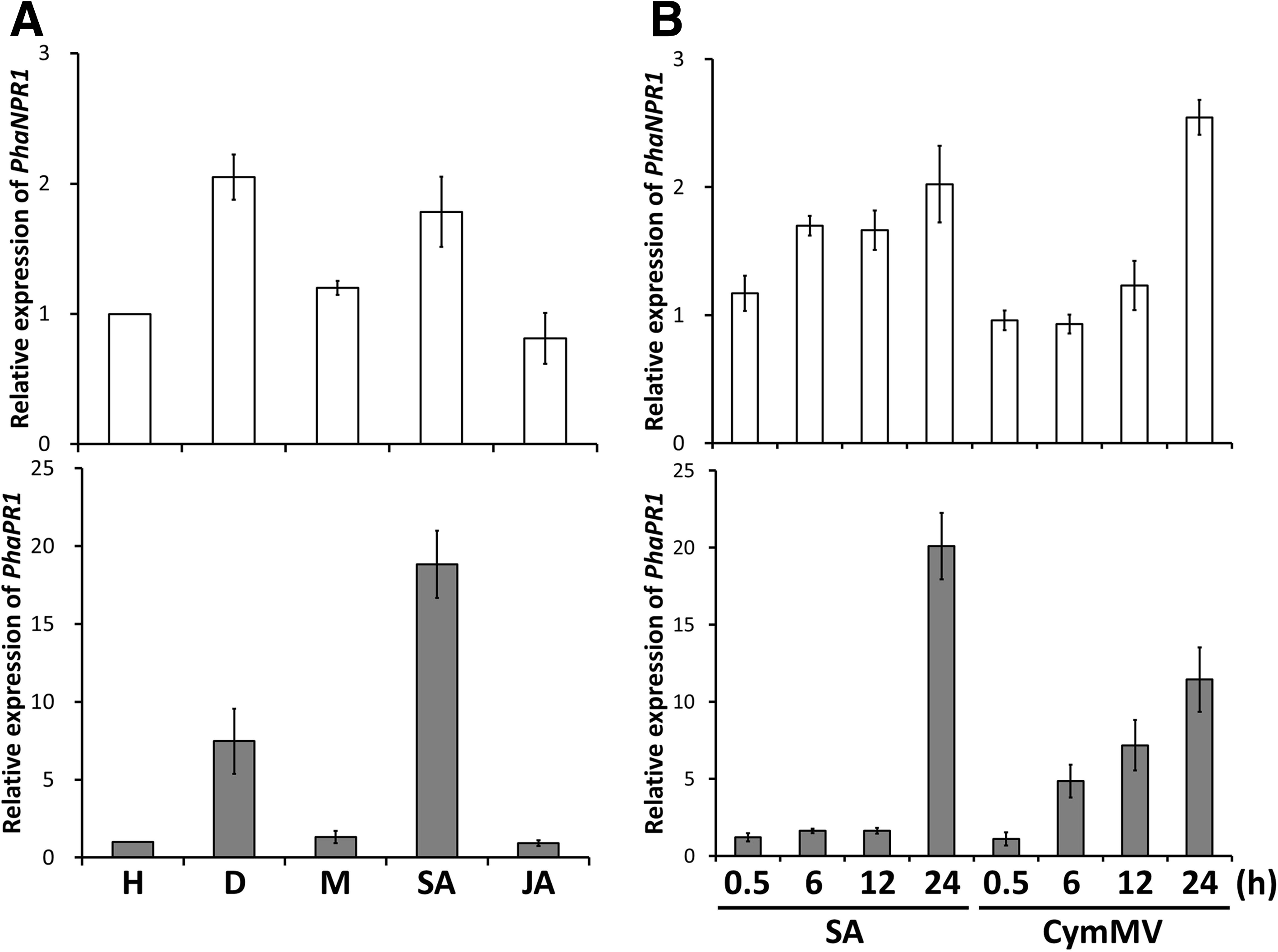

Supplement: Supplementary file 5 — Authors’ original file for figure 4 [file 40529_2013_31_MOESM5_ESM.tif]

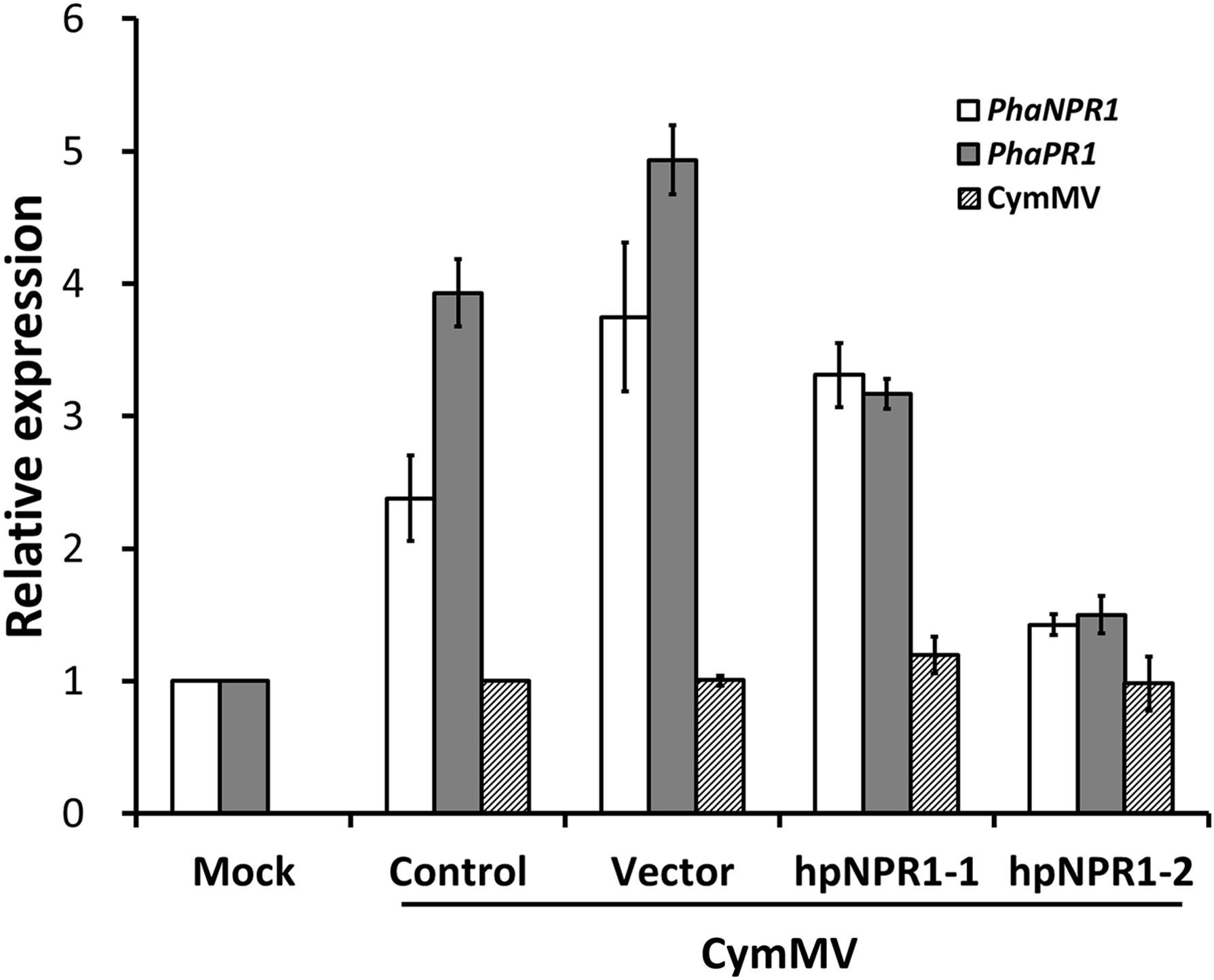

Supplement: Supplementary file 6 — Authors’ original file for figure 5 [file 40529_2013_31_MOESM6_ESM.tif]
