# Supplementary material for: The NPR1 ortholog PhaNPR1 is required for the induction of PhaPR1 in Phalaenopsis aphrodite
Source: Bot Stud. 2013 Sep 6;54:31. doi: 10.1186/1999-3110-54-31 (PMC5432770; doi:10.1186/1999-3110-54-31)
Supplement: Supplementary file 4 — Authors’ original file for figure 3 [file 40529_2013_31_MOESM4_ESM.pdf]

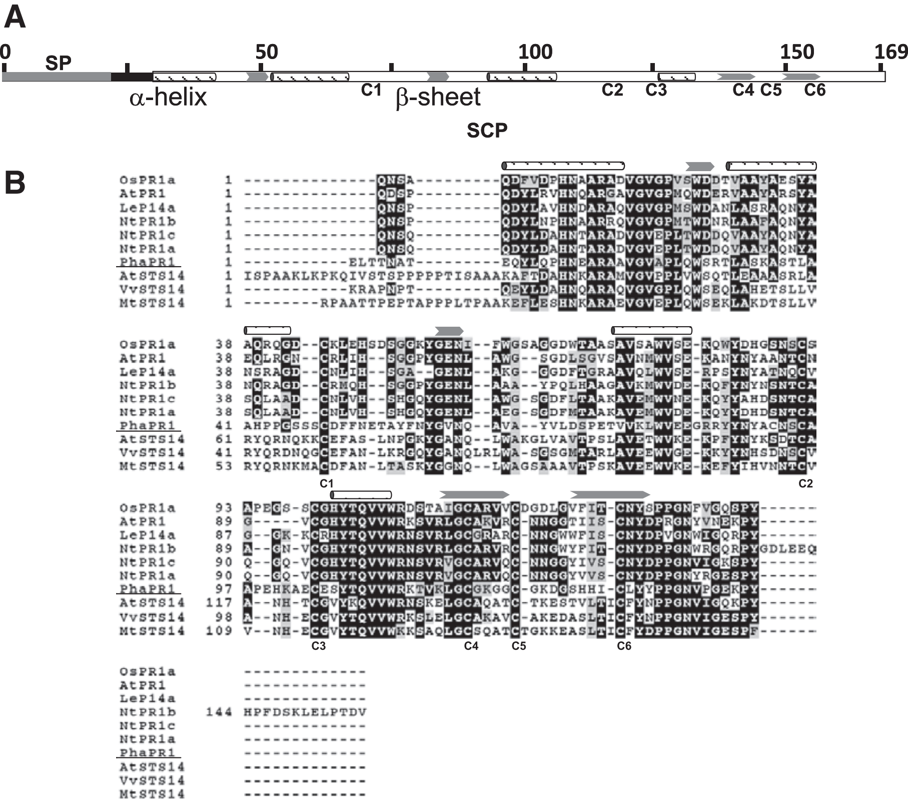
A
0
SP
50
100
150
169
α-helix
C1
β-sheet
C2
C3
C4 C5
C6
SCP
B
OsPR1a 1 --------------QNSA---------QDFVDPHNAARADVGVGPVSWDDTVAAYAESYA
AtPR1 1 --------------QDSP---------QDYLRVHNQARGAVGVGPMQWDERVAAYARSYA
LeP14a 1 --------------QNSP---------QDYLAVHNDARAQVGVGPMSWDANLASRAQNYA
NtPR1b 1 --------------QNSP---------QDYLNPHNAARRQVGVGPMTWDNRLAAFAQNYA
NtPR1c 1 --------------QNSQ---------QDYLDAHNTARADVGVEPLTWDDQVAAYAQNYA
NtPR1a 1 --------------QNSQ---------QDYLDAHNTARADVGVEPLTWDDQVAAYAQNYA
PhaPR1 1 -----------ELTTNAT---------EQYLQPHNEARAAVGVAPLQWSRTLASKASTLA
AtSTS14 1 ISPAAKLKPKQIVSTSPPPPPTISAAAKAFTDAHNKARAMVGVPPLVWSQTLEAAASRLA
VvSTS14 1 -----------KRAPNPT---------QEYLDAHNQARAQVGVGPLQWSEQLAHETSLLV
MtSTS14 1 --------RPAATTPEPTAPPPLTPAAKEFLESHNKARAEVGVEPLQWSEKLAKDTSLLV
OsPR1a 38 AQRQGD--CKLEHSDSGGKYGENI--FWGSAGGDWTAASAVSAWVSE-KQWYDHGSNSCS
AtPR1 38 EQLRGN--CRLIH--SGGPYGENL--AWG--SGDLSGVSAVNMWVSE-KANYNYAANTCN
LeP14a 38 NSRAGD--CNLIH--SGA--GENL--AKG--GGDFTGRAAVQLWVSE-RPSYNYATNQCV
NtPR1b 38 NQRAGD--CRMQH--SGGPYGENL--AAA--YPQLHAAGAVKMWVDE-KQFYNYNSNTCA
NtPR1c 38 SQLAAD--CNLVH--SHGQYGENL--AWG-SGDFLTAAKAVEMWVNE-KQYYAHDSNTCA
NtPR1a 38 SQLAAD--CNLVH--SHGQYGENL--AEG-SGDFMTAAKAVEMWVDE-KQYYDHDSNTCA
PhaPR1 41 AHPPGSSSCDFFNETAYFNYGVNQ--AVA--YVLDSPETVVKLWVEEGRRYYNYACNSCA
AtSTS14 61 RYQRNQKKCEFAS-LNPGKYGANQ--LWAKGLVAVTPSLAVETWVKE-KPFYNYKSDTCA
VvSTS14 41 RYQRDNQGCEFAN-LKRGQYGANQLRLWA-SGSGMTARLAVEEWVGE-KKYYNHSDNSCV
MtSTS14 53 RYQRNKMACDFAN-LTASKYGGNQ--LWAGSAAAVTPSKAVEEWVKE-KEFYIHVNNTCV
C1
C2
OsPR1a 93 APEGS-SCGHYTQVVWRDSTAIGCARVVCDGDLGVFIT-CNYSPPGNFVGQSPY------
AtPR1 89 G-----VCGHYTQVVWRKSVRLGCAKVRC-NNGGTIIS-CNYDPRGNYVNEKPY------
LeP14a 87 G--GK-KCRHYTQVVWRNSVRLGCGRARC-NNGWWFIS-CNYDPVGNWIGQRPY------
NtPR1b 89 A--GN-VCGHYTQVVWRNSVRLGCARVRC-NNGWYFIT-CNYDPPGNWRGQRPYGDLEEQ
NtPR1c 90 Q--GQ-VCGHYTQVVWRNSVRVGCARVQC-NNGGYIVS-CNYDPPGNWRGQRPY------
NtPR1a 90 Q--GQ-VCGHYTQVVWRNSVRVGCARVQC-NNGGYVVS-CNYDPPGNYRGESPY------
PhaPR1 97 APEHKAECESYTQVVWRKTVKLGCGKGGC-GKDGSHHI-CLYYPPGNVPGEKPY------
AtSTS14 117 A--NH-TCGVYKQVVWRNSKELGCAQATC-TKESTVLTICFYNPPGNVIGQKPY------
VvSTS14 98 A--NH-ECGVYTQVVWRKSLELGCAKAVC-AKEDASLTICFYNPPGNVIGESPY------
MtSTS14 109 V--NH-ECGVYTQVVWKKSAQLGCSQATCTGKKEASLTICFYDPPGNVIGESPF------
C3
C4
C5
C6
OsPR1a -------------
AtPR1 -------------
LeP14a -------------
NtPR1b 144 HPFDSKLELPTDV
NtPR1c -------------
NtPR1a -------------
PhaPR1 -------------
AtSTS14 -------------
VvSTS14 -------------
MtSTS14 -------------
